# Tumor angiogenesis, macrophages and mast cell microdensities in endometrioid endometrial carcinoma

**DOI:** 10.3892/ol.2013.1412

**Published:** 2013-06-18

**Authors:** CRISTIANA SIMIONESCU, CLAUDIU MĂRGĂRITESCU, ALEX STEPAN, DANIEL PIRICI, RALUCA CIUREA, NICOLAE CERNEA

**Affiliations:** 1 Departments of Pathology, University of Medicine and Pharmacy of Craiova, Craiova 200349, Romania; 2 Histology, University of Medicine and Pharmacy of Craiova, Craiova 200349, Romania; 3 Obstetrics and Gynecology, University of Medicine and Pharmacy of Craiova, Craiova 200349, Romania

**Keywords:** endometrial carcinoma, tumor angiogenesis, macrophages, mast cells

## Abstract

The present study aimed to observe and compare the values of microvessel density (MVD), mast cell microdensity (McMD) and macrophage microdensity (MphMD) in intratumoral areas compared with the advancing edges, and to assess any correlations between these values and the degree and stage of the neoplasia. The cases of 52 patients who were diagnosed with endometrial carcinoma between 2003 and 2011 were analyzed, the majority of which were in the first stage of the disease (44 cases). Double sequential immunohistochemistry and the hot-spot counting method were used to assess the MVD (CD105^+^ MVD), McMD [tryptase^+^ (Try^+^) McMD] and MphMD (CD68^+^ MphMD) densities. The χ^2^ test, paired Student’s t-test and the Pearson correlation index were used to assess the significance of the results. A weak correlation was observed at the advancing edge only, between CD105^+^ MVD and Try^+^ McMD (P=0.039). No significant differences were identified in the analysis of CD105^+^ MVD, Try^+^ McMD and CD68^+^ MphMD, but wide variations in their distribution were observed. Depending on the tumor stage, CD105^+^ MVD exhibited an intratumoral, indirect correlation with Try^+^ McMD for stage IA (P=0.026) and II (P=0.013) tumors. CD105^+^ MVD presented an indirect correlation with CD68^+^ MphMD in stage IB tumors (P=0.016) and at the advancing edge for well-differentiated tumors (P=0.027). An analysis of the correlation between CD68^+^ MphMD and Try^+^ McMD indicated that the intratumoral levels of CD68^+^ MphMD were directly proportional with the Try^+^ McMD values in well-differentiated (P=0.005) and stage II (P=0.012) tumors, while at the front of the invasion, this correlation was indirect (P=0.010) in stage II tumors. In endometrioid endometrial carcinoma (EEC), angiogenesis is at its most active at the advancing edge of the tumor, where mast cells play a pro-angiogenic role.

## Introduction

Almost 97% of all reported endometrial cancers are classed as carcinomas, which encompass a heterogenous group of tumors that display a range of biological, morphological and pathological characteristics (1–4).

It is well known that tumor transformation and progression do not represent singular events, but involve several steps and complex interactions, including the inflammatory process and angiogenesis, associated with tumors that are establishing a growth-favorable local microenvironment. Macrophages, as a major component of the tumor microenvironment, release growth factors that affect tumor cells or the endothelium of tumor vessels and promote the recruitment of secondary inflammatory cells, including mast cells and neutrophils (5).

In addition, tumor-associated macrophages (TAMs) appear to play a key role in tumor angiogenesis by modulating tumor growth and invasion (6–13). However, mast cells are also known to be able to synthesize and release strong angiogenic cytokines (14), such as tryptase. Tryptase has been reported to be released by mast cells in areas of angiogenesis and also to play a significant role in neovascularization (15).

The aim of the present study was to assess tumor angiogenesis quantified by microvessel density (MVD), mast cell density (McMD) and macrophage density (MphMD), and to assess any putative correlations these factors may have with the tumor stage and grade in patients with endometrioid endometrial carcinoma (EEC).

## Material and methods

### Patients

The cases of 52 patients who were diagnosed with endometrial carcinoma between 2003 and 2011 in the Laboratory of Pathology from the County Emergency Clinical Hospital Craiova (Craiova, Romania) were analyzed. Furthermore, five samples of normal endometrium and myometrium were used as controls. The biological material was obtained from hysterectomy fragments that had been fixed in 10% buffered neutral formalin and then classically processed for paraffin embedding and hematoxylin-eosin staining.

The grade and stage of the neoplasias were used as the morphoclinical parameters and were classified according to the guidelines by the World Health Organization (WHO) (16). In Romania at present, there is no National Register for patients who have been diagnosed with endometrial adenocarcinoma. Approval for the present study was obtained from the ethics committee of the University of Medicine and Pharmacy of Craiova, and written informed consent was obtained from all patients.

### Immunohistochemistry

The panel of antibodies that were used for the immunohistochemical analysis are presented in Table I. The analysis of the morphology, topography and density of the elements of interest was based on sequential double immunohistochemical reactions using the CD105, mast cell tryptase and CD68 antibodies successively. To detect the first antibody, an LSAB2-HRP amplification and detection system (K0675; Dako, Redox Bucharest, Romania) was used with DAB chromogen development (3467; Dako). Detection of the second antibody was performed using a species-specific LSAB2-AP System (K0674; Dako) and chromogen Vulcan Fast Red (FR805S; Biocare Medical, MedicaRom, Bucharest, Romania). An avidin-biotin blocking step was included between the two procedures to mask any available biotin that remained following the first round of the reaction (X0590; Dako). Positive and negative external controls, which omitted the primary antibodies, were also included (data not shown).

The immunohistochemical analysis allowed the visualization of the blood vessels (stained with CD105), mast cells (stained with tryptase) and macrophages (stained with CD68) in all investigated cases. Thus, the McMD, MphMD and MVD in the tumor were determined. Microvascularity was defined as a single endothelial cell or group of endothelial cells that was positive for CD105 and formed a visible lumen that was clearly separated from the adjacent microvessels.

### Microdensity measurement and image acquisition

The microdensity measurements were performed intratumorally and at the advancing edge. The intratumoral area was defined as stromal tissue that contained two or more neoplastic islands, and the invasion front/advancing edge was considered to be a positive structure located on the limit of the tumor-free tissue.

Image acquisition was performed using a Nikon Eclipse E600 microscope, equipped with the Lucia 5 image analysis software (Nikon, Apidrag, Bucharest, Romania).

The microdensities of the elements of interest were determined using the method described by Weidner *et al* (17). The slides were initially scanned at x100 magnification to identify the areas with the highest densities. The quantification was reported as the average value (area or number) for 10 hot-spot fields, with an area of 0.7386 mm^2^, using a 20X objective lens.

To assess the reproducibility of the method, the specimens were counted independently by two observers with a correction factor κ-value of 0.07.

### Statistical analysis

For the statistical analysis, a paired Student’s t-test, one way ANOVA and Pearson’s correlation index were performed using SPSS 10 software (SPSS, Inc., Chicago, IL, USA). The values were reported as mean ± standard deviation. The data averages of the groups were used in order to create data groups and classes for further tests.

## Results

### Normal tissue immunoprofile

The CD105^+^ MVD values in the normal uterus specimens were higher in the endometrium (7.6±2.1; x200 magnification) compared with the myometrium (4.3±1.3; x200 magnification). The analysis of McMD in the normal uterus specimens indicated higher average values in the myometrium (9.7±1.7; x200 magnification) compared with the endometrium (3.7±1.3; x200 magnification), while MphMD had higher values in the endometrium (93±43.7; x200 magnification) compared with the myometrium (3.2±1.2; x200 magnification).

### Clinical data

The average age of the patients with endometrioid carcinoma was 57.8 years (range, 43–75 years). In terms of the degree of differentiation, the well- and moderately-differentiated tumors of stages IA (17 cases) and IB (27 cases), respectively, were most prevalent, as the majority were diagnosed in the early stages.

### Immunohistochemistry

The analysis of the CD105^+^ MVD, Try^+^ McMD and CD68^+^ MphMD values revealed no significant differences, but there were variations in their distribution when the data were grouped into tumor topography (intratumoral vs. advancing edge), grade and tumor stage subcategories.

**Figure 1.**
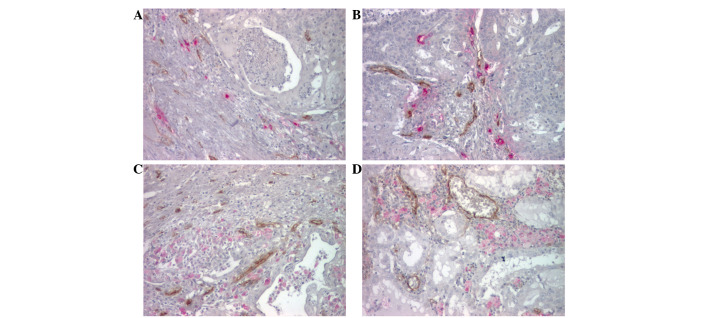
Endometrial carcinoma. Mast cells and microvessels from (A) the invasion front and (B) the intratumoral area; double immunohistochemistry for tryptase (AP-Fast Red; red and CD105 (HRP-DAB; brown). Macrophages and microvessels from (C) the invasion front and (D) intratumoral area; double immunohistochemistry for CD68 (AP-Fast Red; red) and CD105 (HRP-DAB; brown). Magnification, x100.

The morphology and topography of the vessels that were stained using CD105 indicated the presence of numerous vascular structures, which were more concentrated at the advancing edge (19.9±6.3; x200 magnification) compared with the intratumoral areas (12.8±4.6; x200 magnification). The vessels from the advancing edge had significantly larger lumens, and were more branched and dilated compared with the intratumoral vessels, which were less branched with a collapsed lumen (Fig. 1).

The analysis of the Try^+^ mast cells revealed that they were present in all the studied cases, with various shapes, sizes, degrees of degranulation and aspects of mastocytoclasia. Overall, the mast cells were located mainly around the vessels (Fig. 1A and B), isolated or associated with the inflammatory site, and were more numerous at the advancing edge (7.4±4.5; x200 magnification) compared with the intratumoral location (4.7±3.8; x200 magnification).

The CD68^+^ macrophages were also present in all cases studied, with a dendritic morphology, abundant cytoplasm and round-oval nuclei. Variable densities of the macrophages were observed, being more numerous in the intratumoral areas (11.2±9.2; x200 magnification) and predominantly perivascular (Fig. 1C and D) or adjacent to areas of necrosis compared with the advancing edge of the tumor (8.3±10.2; x200 magnification).

The data from the immunohistochemical analysis for the mean microdensities of the morphological elements of interest, depending on the grade and tumor stage, are presented in Table II.

### Clinico-morphological correlations

**Figure 2.**
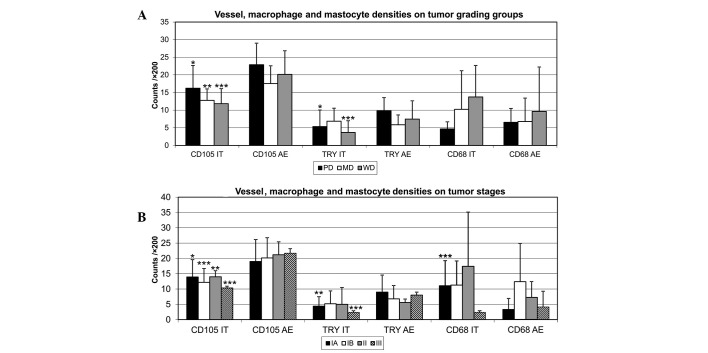
The distribution of vessels (CD105^+^), macrophages (CD68^+^) and mast cells (TRY) depending on tumor (A) differentiation degree and (B) stage. TRY, tryptase; IT, intratumoral; AE, advancing edge; WD, well-differentiated; MD, moderately-differentiated; PD, poorly-differentiated. Error bars represent standard deviation. ^*^P<0.05; ^**^P<0.01; ^***^P<0.001.

According to the histological grade of the analyzed endometrioid carcinomas, the Student’s t-test indicated that CD105^+^ MVD for all degrees of differentiation and Try^+^ McMD for well- and poorly-differentiated tumors had significantly lower intratumoral values compared with the advancing edge (Fig. 2A).

The analysis of CD105^+^ MVD, Try^+^ McMD and CD68^+^ MphMD, depending on the tumor stage using a Student’s t-test, revealed that the values of CD105^+^ MVD for all stages and of Try^+^ McMD for stages IA and III, were lower intratumorally compared with the advancing edge (Fig. 2B). In contrast, the CD68^+^ MphMD values in stage IA tumors showed significant differences and were higher intratumorally compared with the advancing edge.

Overall, using Pearson’s test, CD105^+^ MVD indicated a weak correlation with Try^+^ McMD [r(50)=0.287, P=0.039] at the advancing edge of the tumor.

When considering the data grouped into tumor stages, Pearson’s test indicated that CD105^+^ MVD exhibited an intratumoral, indirect correlation with Try^+^ McMD stage IA [r(15)=−0.538, P=0.026] and II [r(3)=−0.951, P=0.013] tumors. CD105^+^ MVD also presented an indirect correlation with CD68^+^ MphMD at the advancing edge for well-differentiated forms [r(28)=−0.403, P=0.027] as well with lesions from stage IB tumors [r(25)=−0.458, P=0016].

Pearson’s test showed that CD68^+^ MphMD varied in direct correlation with Try^+^ McMD in well-differentiated [r(28)=0.502, P=0.005] and stage II [r(3)=0.952, P=0.012] tumors. In contrast, at the advancing edge of the stage II tumors, this correlation was indirect [r(3)=−0.959, P=0.010].

## Discussion

Angiogenesis and inflammation have numerous common pathways, as they are biological processes that are closely associated with cancer. Angiogenesis is critical for the continuous growth of tumors and the development of metastases (18). The initiation of angiogenesis is primarily regulated by the balance between pro- and anti-angiogenic factors, and represents an early and essential event in tumor development and progression (19,20). A significant role in this process appears to be played by the interaction between tumor cells and the tumor microenvironment, which indirectly contributes to the induction of angiogenesis.

Macrophages and mast cells are pivotal inflammatory cells in the tumor stroma, and are present in the majority of malignant neoplasms. In the second half of the 1990s, a correlation was identified between mast cells and angiogenesis in various malignancies (21). Mast cell tryptase was shown to be a potent angiogenic factor (15). Furthermore, several studies have demonstrated the association between the intensity of tumor vascularization and macrophage infiltration in numerous cancers, including endometrial cancer (22), suggesting that TAMs increase the angiogenic potential of tumors by producing pro-angiogenic factors, including vascular-endothelial growth factor (VEGF) (23).

In the present study, certain differences were identified between the distribution of CD105^+^ MVD and Try^+^ McMD in relation to the tumor grade and stage. CD105^+^ MVD for tumors of all degrees of differentiation, and Try^+^ McMD in well- and poorly-differentiated tumors, had significantly lower intratumoral values compared with the advancing edge. Depending on the tumor stage, CD105^+^ MVD for all stages and Try^+^ McMD for stages IA and II were lower intratumorally compared with the advancing edge. Overall, the Pearson correlation test indicated the existence of a weak correlation between CD105^+^ MVD and Try^+^ McMD levels in the advancing edge. In contrast, the analysis of the correlation with regard to the tumor stage between CD105^+^ MVD and Try^+^ McMD indicated an intratumoral, indirect correlation for stage IA and II tumors. Although angiogenesis, quantified by CD105^+^ MVD and Try^+^ McMD, was more active at the advancing edge, only a weak statistical correlation was observed between the two parameters. This would argue in favor of the pro-angiogenic effect of mast cells in the advancing edge of endometrial carcinomas.

Certain studies have indicated the existence of a correlation between MVD and McMD and the degree of differentiation of endometrial carcinomas (24), while others have recorded its absence (25–27).

Ribatti *et al* (24) report that endometrial carcinoma angiogenesis, measured by the number of CD31^+^ microvessels, is strongly correlated with the number of Try^+^ mast cells, with poorly-differentiated tumors containing a higher number of vessels compared with well-differentiated tumors. In contrast, no correlation was observed in other studies between the density of C-kit^+^(26) or try^+^(27) mast cells or the number of CD31^+^ microvessels and the tumor grade. Gosku *et al* (25) reported a more intense vascularity in the tumor stroma and myometrium of high-grade endometrial cancers compared with low-grade tumors, and also that try^+^ mast cells do not increase in parallel with the histological grade of tumors. The lack of correlation between mast cell density, angiogenesis and the histological tumor grade seem to suggest that mast cells are not a significant prognostic factor in tumor progression (25).

Data obtained from the literature on the correlation between mast cells density and tumor stage are also contradictory. Ribatti *et al* (24) reported that angiogenesis and try^+^ mast cell number increases with tumor progression. Similarly, Cinel *et al* (26) indicated a statistically significant correlation between a high density of C-kit^+^ mast cells and the presence of myometrial invasion, with high levels in 54% of the analyzed cases, 94% of which were also associated with myometrial invasion. In contrast, other studies have reported no correlation between Try^+^ McMD and myometrial invasion (25,27).

In the present study, similar to the Try^+^ McMD analysis, the test for the CD68^+^ MphMD-grouped data showed certain differences with regard to the tumor stage. The levels of CD68^+^ MphMD were higher intratumorally than in the advancing edge in stage IA tumors. With regard to the association between CD105^+^ MVD and CD68^+^ MphMD, the existence of certain indirect correlations at the advancing edge for well-differentiated forms, as well as for stage IB tumors, were identified. Overall, the lack of correlation between CD105^+^ MVD and CD68^+^ MphMD values would indicate a minor role of these cells in the angiogenesis process.

In the case of macrophages, data from various studies with regard to the correlation between the tumor grade and stage are also controversial. Espinosa *et al* (28) reported high CD168^+^ macrophage infiltration in high-grade endometrioid carcinomas and the presence of a greater number of CD31^+^ vessels compared with low-grade endometrioid tumors. Hashimoto *et al* (22) reported that TAMs from tumor nests and stroma were significantly increased in high-grade tumors. A univariate analysis by Soeda *et al* (29) observed that a statistically significant correlation existed between the number of intratumoral TAMs and the tumor grades defined by the International Federation of Gynecology and Obstetrics (FIGO), and also between the number of intratumoral TAMs and MVD. Another study has shown that the moderate or strong expression of VEGF is significantly associated with a high MVD and an increased number of CD68^+^ macrophages for aggressive tumor subgroups (30). The literature reports a correlation between macrophages and myometrial invasion, but not as a prediction factor for prognosis (22,28,29).

Certain studies have provided statistically significant results for macrophage infiltration and myoinvasive vs. non-myoinvasive tumors (28) and the depth of myometrial invasion (22,29), suggesting that tumor angiogenesis is triggered and enhanced by stromal macrophages, which regulate the progression of endometrial carcinoma (28). Ohno *et al* (31) reported that only the CD68^+^ macrophages from within the areas of necrosis were associated with the clinical stage and level of myometrial invasion, while Soeda *et al* (29) identified this correlation for the CD68^+^ macrophages in the intratumoral and advancing edge areas.

In the present study, Try^+^ McMD and CD68^+^ MphMD at the intratumoral level were correlated in well-differentiated and stage II forms. At the advancing edge, there was an indirect correlation in stage II tumors. Although there were no significant correlations between McMD and MphMD, an indirect correlation was observed between the two parameters in the stage II tumors at the advancing edge, and a direct correlation intratumorally. This would emphasize the dual agonist-antagonist roles of the two cell populations in the processes of angiogenesis and tumor progression in endometrial carcinoma.

The existence of conflicting results in the literature is most likely to be caused by the large variation in tumor types and stages, the location of the inflammatory cells and the methods used to label the inflammatory cells and blood vessels, in addition to the lack of a standardized assessment of angiogenesis or the inflammatory cells (25,32). A significant limitation is the variation in the methods for reporting the topography of the mast cells and macrophages (31). In addition, labeling the vessels for CD31 (24,25,27,28) or CD105 (33,34), the mast cells for tryptase (24,25,35), toluidine blue (27) or C-kit (26) and the macrophages for CD68 (29–31) or CD163 (28) may cause discrepancies.

In conclusion, angiogenesis was observed to be more intense in the advancing edge, where it showed a weak correlation with Try^+^ McMD. The correlation between CD68^+^ MphMD and angiogenesis was indirect and only identified at the intratumoral level in stage IA and II tumors. A direct correlation existed between Try^+^ McMD and CD68^+^ MphMD at the intratumoral level in well-differentiated and stage II tumors, while in the advancing edge, this correlation was indirect and only identified in stage II tumors. The present data require a quantification of angiogenesis and McMD and MphMD in endometrial carcinomas, in order to determine the prognosis and future development of alternative therapies that may target these denominators, with potential benefits for patients.

## Figures and Tables

**Table I.** Panel of antibodies used for the immunohistochemistry.

Antibody	Clone, manufacturer	Dilution	Antigenic retrieval	External positive control
CD105 (endoglin)	Polyclonal, Thermo Scientific	1:50	Citrate, pH 6	Kidney
Mast cell tryptase	AA1, Dako	1:100	Citrate, pH 6	Skin
CD68	KP1, Dako	1:50	Citrate, pH 6	Skin

Thermo Scientific (Motortech, Timisoara, Romania); Dako (Redox Bucharest, Romania).

**Table II.** Correlations of MVD, McMD and MphMD in EEC with clinico-morphological investigated parameters.

Parameter	Tumoral degree			Tumoral stage			
	
	WD	MD	PD	IA	IB	II	III
No.	30	13	9	17	27	5	3
MVD							
IT	11.8±4.2	12.8±3.2	16.2±6.4	13.9±5.6	12.2±4.5	14.0±2.0	10.3±0.6
AE	20.1±6.7	17.5±5.0	22.8±6.1	19.0±7.2	20.1±6.6	21.2±4.2	21.7±1.5
P-value	1.938×10^−7^	0.004	0.019	0.014	1.875×10^−6^	0.004	0.000
McMD							
IT	3.7±3.3	6.8±3.7	5.3±4.7	4.4±3.1	5.2±4.2	5.0±5.5	2.3±0.6
AE	7.5±5.2	5.8±2.8	9.7±3.8	9.0±5.6	6.8±4.4	5.6±1.1	8.0±1.0
P-value	0.000	0.221	0.020	0.002	0.088	0.408	0.000
MphMD							
IT	13.7±8.9	10.2±10.9	4.6±2.0	11.0±8.2	11.3±7.9	17.4±17.7	2.3±0.6
AE	9.6±12.6	6.8±6.6	6.5±3.9	3.2±3.6	12.3±12.5	7.2±5.2	4.0±5.2
P-value	0.075	0.169	0.108	0.000	0.358	0.125	0.305

EEC, endometrioid endometrial cancer; MVD, microvessel density; MphMD, macrophage microdensity; McMD, mast cell microdensity; IT, intratumoral; AE, advancing edge; WD, well-differentiated; MD, moderate-differentiated; PD, poor-differentiated. P-values were obtained by Student’s t-test. All data, with the exception of No., are presented as mean ± standard deviation.
